# A hybrid solution for extracting structured medical information from unstructured data in medical records via a double-reading/entry system

**DOI:** 10.1186/s12911-016-0357-5

**Published:** 2016-08-30

**Authors:** Ligang Luo, Liping Li, Jiajia Hu, Xiaozhe Wang, Boulin Hou, Tianze Zhang, Lue Ping Zhao

**Affiliations:** 1 LinkDoc Inc, 8 Haidian Street, Block A, 8th Floor, Haidian District, Beijing, China; 2 Division of Public Health Sciences, Fred Hutchinson Cancer Research Center, Seattle, WA USA

**Keywords:** Big data, Big medical data, Clinical research, Clinical decision support system, Cloud-based system, Double data entry, Electronic medical record, Health service research, Structured data and unstructured data

## Abstract

**Background:**

Healthcare providers generate a huge amount of biomedical data stored in either legacy system (paper-based) format or electronic medical records (EMR) around the world, which are collectively referred to as big biomedical data (BBD). To realize the promise of BBD for clinical use and research, it is an essential step to extract key data elements from unstructured medical records into patient-centered electronic health records with computable data elements. Our objective is to introduce a novel solution, known as a double-reading/entry system (DRESS), for extracting clinical data from unstructured medical records (MR) and creating a semi-structured electronic health record database, as well as to demonstrate its reproducibility empirically.

**Methods:**

Utilizing the modern cloud-based technologies, we have developed a comprehensive system that includes multiple subsystems, from capturing MRs in clinics, to securely transferring MRs, storing and managing cloud-based MRs, to facilitating both machine learning and manual reading, and to performing iterative quality control before committing the semi-structured data into the desired database. To evaluate the reproducibility of extracted medical data elements by DRESS, we conduct a blinded reproducibility study, with 100 MRs from patients who have undergone surgical treatment of lung cancer in China. The study uses Kappa statistic to measure concordance of discrete variables, and uses correlation coefficient to measure reproducibility of continuous variables.

**Results:**

Using the DRESS, we have demonstrated the feasibility of extracting clinical data from unstructured MRs to create semi-structured and patient-centered electronic health record database. The reproducibility study with 100 patient’s MRs has shown an overall high reproducibility of 98 %, and varies across six modules (pathology, Radio/chemo therapy, clinical examination, surgery information, medical image and general patient information).

**Conclusions:**

DRESS uses a double-reading, double-entry, and an independent adjudication, to manually curate structured data elements from unstructured clinical data. Further, through distributed computing strategies, DRESS protects data privacy by dividing MR data into de-identified modules. Finally, through internet-based computing cloud, DRESS enables many data specialists to work in a virtual environment to achieve the necessary scale of processing thousands MRs within days. This hybrid system represents probably a workable solution to solve the big medical data challenge.

**Electronic supplementary material:**

The online version of this article (doi:10.1186/s12911-016-0357-5) contains supplementary material, which is available to authorized users.

## Background

In medical practice, healthcare workers, including physicians, nurses and supporting staff, produce a great amount of clinical and administrative data, from general check-up information, drug prescription information, treatment records, physicians’ notes, laboratory results/images, surgical information, in addition to financial or administrative information [1]. Most of clinical data are traditionally stored in legacy systems, including paper-based filing system [2, 3], and are now increasingly stored in electronic medical record system (EMR) on computers [1, 4, 5]. Accumulations of clinical data have produced “big biomedical data” (BBD). Successful exploration of big data sets in industries and ecommerce [6–9] prompts healthcare professionals to seek research opportunities within medical big data as well. For example, one may be interested in research questions such as which treatments are more effective than others, whether or not new or existing therapies are safe in real world practice, what is the cost-effectiveness of many equivalent treatments, how we learn about and streamline clinical practices, and how we develop clinical decision support to improve clinical diagnosis and management [10–12]. Collectively, there is a high hope for the secondary use of BBD for clinical and healthcare research [13, 14]. Conceptually, it seems straightforward to organize all available clinical data into a database, after linking different pieces of data sets via patient identification numbers.

In practice, there are many challenges facing the big data research in healthcare. Obviously, many challenges are associated with data privacy, data ownership, data security, interdisciplinary collaborations and conflicts of research and commercial interests. Here we focus on one specific technical challenge on how to extract “computable data elements” from BBD and to store them into one database. Since most BBD data sets are created for operational purposes, they are largely unstructured, i.e., many texts and drawings document symptoms, diagnoses, laboratory test results, or physicians’ notes. In some cases, BBD include anatomic hand-drawings with informal annotations. Such MRs are highly informative for trained physicians, but unfortunately not machine-readable, nor computable. Hence, such BBD, regardless how large they are, are not readily available to fulfill the promises of the big data.

In the biomedical community, researchers and physicians have long realized that BBD provides valuable clinical information for research, and have been using BBD for various research projects [15]. Unless working with clinical research database with structured data elements, clinical researchers typically have to review all medical charts, and to extract manually pertinent clinical data to address specific clinical research questions, i.e., manually converting unstructured MRs into semi-structured data that are appropriate for specific research questions on hand. While it is probably the practical method in routine use to extract purpose-driven information from selected MRs, this manual extraction is unfortunately not scalable to deal with general BBD, because of unattainable highly skilled human resources, i.e., physicians. An alternative approach, commonly adopted in clinical trials, is to design specific forms, such as Case Report Form (CRF) as a way of identifying cases [16, 17]. Typically, CRF is simple, and includes only limited structured variables so that nurses can easily fill in CRF forms, as a task to their already busy routines. After receiving filled CRFs, research staff enter structured CRF data elements into database via a double-entry system [18]. Even though CRFs are useful for identifying and recruiting patients, they are of limited use for general clinical researches, beyond the tailored clinical trial. Hence, the data extraction method used in clinical trials is not readily applicable to process BBD either.

To solve this data extraction challenge, some computer scientists have placed high hope on machine-learning algorithms, data mining or artificial intelligence, based on recently developed Natural Language Processing (NLP) and Text Predictics [19–29]. Conceptually, if these novel machine-learning algorithms could process natural spoken language, one would hope to utilize these technologies to read unstructured data in BBD, to comprehend the intent of physicians, to quantify research information, and to create a structured database [28]. In practice, numerous complications may distort machine-reading of unstructured MRs at this time. For example, algorithms and predictive models depend on the availability of well annotated large training data sets, with methods such as neural networks, cluster analysis, genetic algorithm, decision trees and support vector machines to find hidden patterns. For these methods to perform well, one needs to have a manual curated “training set” with gold standard, which is not easily attainable. On the other hand, NLP based on machine learning algorithms intends to interpret natural spoken language based on established ontologies, and is making steady but slow progresses towards high accuracy. At this time, the accuracy is not high enough for medical uses. Nevertheless, it remains a hope that one day, we can combine machine-learning algorithms, statistical learning methods, and linguistic knowledge, to predict structured data elements from unstructured data, as evidenced by some pioneering studies [29]. When such methodologies will become available, we will be able to extract accurate structured data automatically from MRs in the future.

Waiting for a better solution to exploring MR is not an option, in light of urgent needs for better-personalized treatment and improved patient-centered outcomes at affordable costs, especially for many developing countries. Here we propose an interim solution to extract structured data from unstructured MRs, based on our intimate understanding of EMRs and needs of clinical research. Specifically, this manuscript describes a double-reading/entry system (DRESS) for extracting structured elements from unstructured MRs, using power of both information technologies and human intelligence. Utilizing the “divide and conquer” strategy, DRESS separates a target MRs into relatively homogeneous modules with varying levels of required medical knowledge. On each module, trained and certified personnel with sufficient background for the corresponding module and is known as “Data Specialist” (DS), reads corresponding module in MRs, and extracts pertinent data into the database system. To control quality of reading comprehension and data entry, DRESS requires two independent entries by DS. Discrepancies in entered information are then subject to the adjudication by third and senior DS. In essence, DRESS represents a hybrid system of manual curation and computer automation.

## Methods

### DRESS overview

We develop DRESS as a general methodology for extracting structured data elements from MRs. For simplicity in description, we focus the presentation on a DRESS application to processing MRs created in surgical departments dealing with cancer patients. Suppose that we are building a BBD for multiple oncology departments in hospitals located in diverse geographic areas (using one common language, i.e., Chinese in DRESS). As expected, these oncologic departments in different hospitals may use diverse systems for storing clinical data, from legacy system to EMR. For a coherent discussion, we suppose that all hospitals use legacy systems with diverse paper formats. Clinical data recorded on papers include the following structured data: patient’s demographics (birth date, birth place, ethnicity, blood type), admission/discharge information (dates, attending physicians, costs), medical history, surgical operational details, pathology, image data, clinical examination data, laboratory test data, uses of medications, chemo/radio therapies, genetic testing results, clinical assessments at discharge, and follow-up information.

Clinical data collected from individual hospitals and stored in our database cannot be used for clinical research, without official approval of respective Institutional Review Board (IRB). Just as in United States, each major healthcare organization or medical university has IRB, and their rules and regulations are locally developed and enforced. Hence, when launching a multi-center study with clinical data from our database, investigators need to apply an approval for accessing and using their own clinical data to participate in the multi-center study.

Recognizing that cancer treatment is specific to anatomic sites with specialized procedures and that recorded information in MRs varies between hospitals, we devote a substantial amount of time and effort to create a common data model (CDM) for each cancer site, e.g., lung cancer is focused in this discussion. For this development, we organized an interdisciplinary team of oncologists with pertinent specialty, experienced staff with clinical data collection and experienced computer scientists familiar with medical ontologies. Through iterative trial-error process, the team comes up a general CDM for collecting structured data elements for patients who undergo treatment of lung cancer. The current version of CDM is LinkDoc-CDM (version2.1.12), created on November 27, 2015. Then, working with individual hospitals, we identify all pertinent data elements from hospital-specific MRs, and convert them to necessary data elements by CDM. Hence, after all MRs are processed and organized into our system, the resulting data elements in the database by the same CDM.

**Fig. 1:**
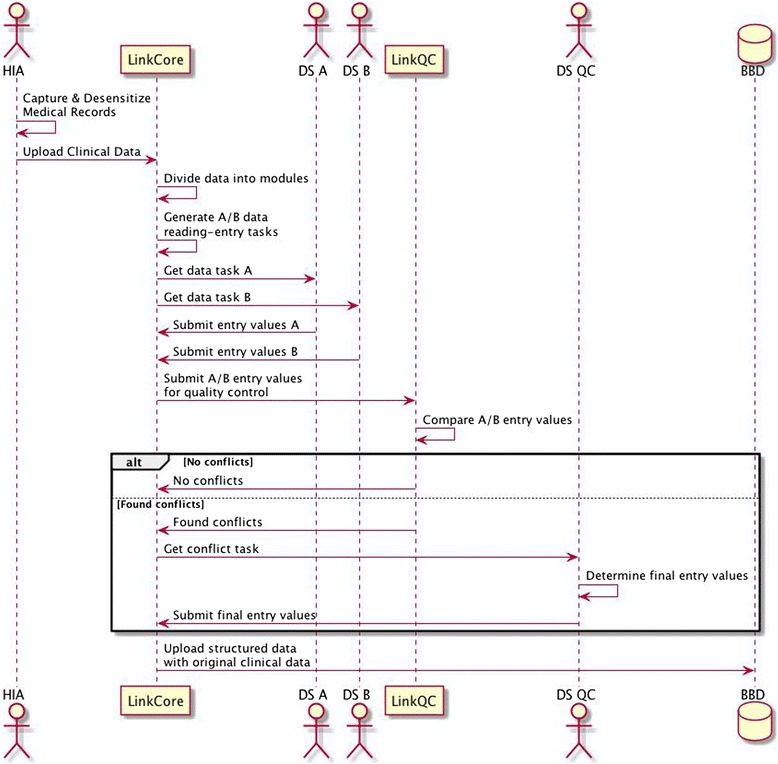
An overview of DRESS from accessing clinical data in legacy system, to de-sensitizing all clinical data, to create double reading-entry tasks, to perform quality control, and ultimately to store the structured data into BBD

DRESS fundamentally represents a hybrid system with manual curations and with information technologies. A pre-requisite to developing DRESS is for us a database developer to recognize that healthcare workers generate MRs for delivering healthcare, and their priority is to ensure patient-centered services and outcome, rather than data collection and quality. Often, healthcare workers are too busy to devote much attention to collecting data, even though all of them recognize the importance having MRs for downstream clinical research. As part of DRESS, we assign one college-educated personnel, typically, graduates from nursing schools, to each oncologic department, to work side-by-side with healthcare workers, and call him/her a health-information assistant (HIA). The primary responsibility is for HIA to gather all pertinent MR, both from clinics and from hospital EMR. To enable the HIA to efficiently work with healthcare workers, we develop a LinkMR (abbreviated from Linking Medical Records), a subsystem with specific functionalities, i.e., to assign an internal identification number for the patient, to remove patient-specific and doctor-specific identification information, to scan paper-based MR into images, and to upload images to a cloud-based data storage, representing the first step of DRESS (the first column in Fig. 1). The second subsystem in DRESS is LinkCore (an abbreviation from Linkage Core), to divide uploaded MR images into different modules through subsystem (the second column in Fig. 1), and to create data entry tasks for DS and to coordinate quality control with data specialist for quality control (DS-QC). The third subsystem is LinkQC (abbreviated from Linking Quality Control) (the fifth column in Fig. 1), to compare double entries by two DSs for quality control, to resolve conflicts through quality control specialists, and to carry out other quality control activities.

### Subsystem: LinkMR

**Fig. 2:**
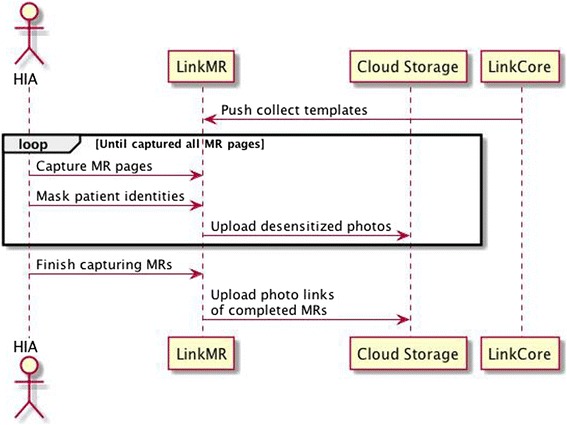
LinkMR, operated on either iPAD or PC, scans in medical records, processes them by OCR, divides them into modules, and submits encrypted MR images to cloud storage

As noted above, HIA is working side-by-side with healthcare workers (physicians, nurses and staff) in the clinical setting. Meanwhile, all clinical data, collected from clinics, are strictly confidential. To balance the effectiveness of gathering clinical data and the necessity of protecting patient/physician’s privacy, we develop the LinkMR system of DRESS on iOS system so that HIA can run LinkMR on iPad or iPhone (Fig. 2). The mobile devices scan MRs, images and other clinical data into digital images. After receiving data, LinkMR carries out “optical character recognition” (OCR), identifying personal information, such as name, social security ID, address, etc., and assigns internal study ID. Note that internal identification number is chosen to be, if available, the official “social security ID” issued by government and is commonly used on MR in China. In the event that the social security ID is not available, the internal identification number is constructed as a composite of “patient’s name”, “hospitalization record number”, and “hospital identification number”. Automatically, LinkMR creates a separate linkage file for study ID and personal information. After stripping away personal and identification data and creating a de-identified data file, LinkMR uploads these images to a centrally controlled LinkCore system (see next section) via a secured private cloud. Such images will be organized (see the following) ready for manual reading. While mobile-based LinkMR offers the maximum flexibility for HIA to work in computer-unfriendly environments, we have also implemented LinkMR in Window environment, so that HIA can process clinical data more efficiently on personal computers than mobile devices. Further, LinkMR operates the designated scanner that uptakes more clinical data with better quality. Indeed, uploading scanned MR images to cloud is much more efficient via PC than via mobile devices. Once all scanned clinical data are in cloud storage, LinkCore system takes on all database management, data security protocol and protection of personal privacy.

When designing this system, we were confronted with a key choice between private cloud storage and private server farm, from the perspective of the data security. Indeed, if financial resources were of no concern, one could argue that private server farm would offer better data security than cloud storage, since the latter involves the third party. However, recent technological improvement in clouding computing and storage has gradually chipped away the superiority of private server farm with respect to data security. Increasingly, IT community in China, following the trend of United States in adopting cloud computing, begins to coalesce around cloud computing for storing governmental data as well as for clinical data. In the current implementation, we use cloud storage and computing capacity on Alibaba cloud (https://intl.aliyun.com). Through this arrangement, we were able to devote more resources to create data security protocols and policies, while spending limited resources on data storage hardware. Because of our direct experiences working on healthcare data in cloud, LinkDoc, teamed up with Alibaba, has been commissioned to create China standard on data security policies for National Health and Family Planning Commission of China.

### Subsystem: LinkCore

LinkCore manages all de-identified clinical data in the computing cloud storage. By storing all de-identified clinical data in the cloud, LinkCore provides a cost-effective management of sensitive clinical data, and is a scalable solution for fast-growing data without large investment into hardware infrastructure and associated delay. Utilizing PHP [30] and running on CentOS Linux system [31], LinkCore takes in unstructured and de-identified clinical data, processes all data, and stores the structured data in MongoDB (https://www.mongodb.org). The primary reason for choosing

MongoDB is that it is flexible for complex data of any structures without schema-restrictions, and for rapid iterations to improve data business models.

**Fig. 3:**
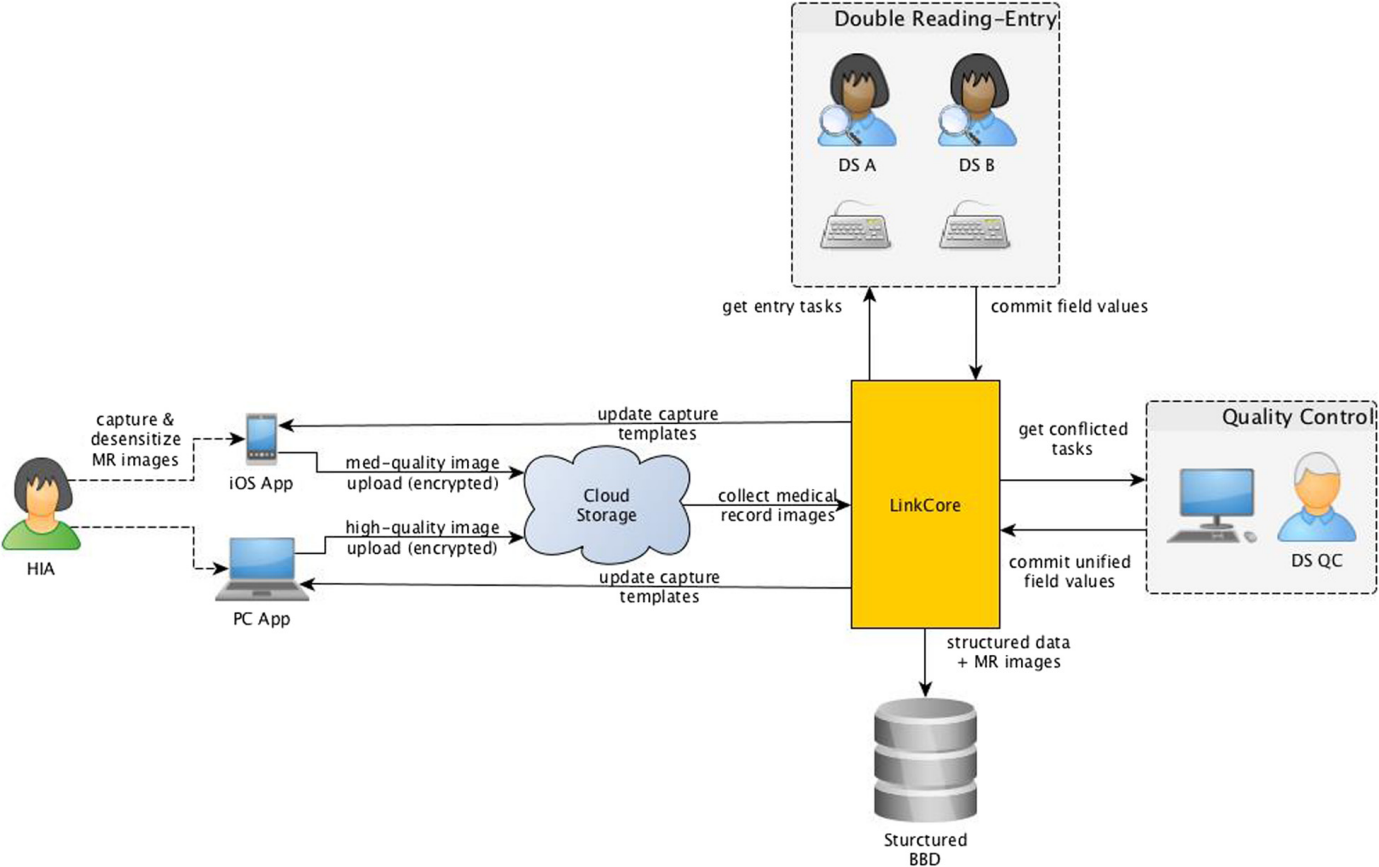
LinkCore takes in unstructured clinical data from HIA, divides clinical data into multiple modules, assigns data to DS A and B, performs LinkQC to identify conflicts, and allows DS QC to make final adjudicated values on those conflicts

Now from the perspective of LinkCore, it places a central role of interacting with HIA, mobile-based or PC-based App equipment, cloud storage of all clinical data, DS, quality controller, and, cloud storage of all structured clinical data (Fig. 3). The administrator of LinkCore distributes data capture templates to HIA in each hospital. Once clinics authorize use of specific batch of clinical data, HIA uploads de-identified clinical data to LinkCore via iOS-based or Window-based apps.

After receiving de-identified clinical data in the cloud, LinkCore divides clinical data into different modules. Each module has relatively homogeneous contents within certain subject matters. Some modules require collection of data elements that can be directly extracted from MR, and other modules may require “searching” and “human interpretations”. All modules are thus assigned varying degrees of complexity, based on the required human interpretations. LinkCore makes these modules available for eligible DS to select data-entry tasks appropriate for their certified levels (Fig. 3). In addition, LinkCore identifies the priorities for various tasks and potential order of task completions.

As noted above, many hospitals have their own forms and organizations of MRs. As part of the certification process, an experienced staff with clinical background and with hospital-specific MRs will develop hospital-specific guidelines, and will provide training to DS prior to exposing those DS to associated MRs. Training and certification are continuing processes as DRESS takes MRs from newly participating hospitals.

It is also worth noting that DS plays a special role in DRESS. As we know, reading and extracting information from typical MRs is complex and generally requires knowledges of multiple disciplines, possessed by physicians who receive years of training and clinical practice. Through dividing entire MR into multiple relatively homogeneous modules, DRESS presents modules appropriate to DS who have varying degrees of experiences and knowledges. Some modules require DS to be knowledgeable about specific medical procedures, while other modules require DS to have good judgement or to be detailed. Based on experiences, we have found that college graduates, preferably health-related education, are ideal candidates for DS positions. Currently, 25 % of DS working on DRESS are graduates from nursing schools.

Typically, we have two DS, call them DS A and B, to perform data-reading and data-entry tasks and to submit structured data via LinkCore.. Afterwards, LinkCore calls on LinkQC system (see next section) to detect possible conflicts between the two independent entries. When quality issues are present, LinkCore archives them to a pool of quality control tasks and prompts them to DS-QC (Fig. 3). The responsible DS-QC reads the unstructured clinical data (in the scanned images of MR), determines post-quality control values for those in conflict. Following the initial adjudication, DS-QC sends this query back to DS A and B without disclosing post-quality control values, and ask the DS in question to repeat the data entry, as another quality control on the DS-QC. Once the consensus is reached, DS-QC will commit adjudicated values into the final BBD database system. However, if a DS questions the validity of values entered by DS-QC, the DS can escalate this case to higher level of quality control by a senior DS-QC who will then be making a final decision on the correct value.

### Subsystem: LinkQC

**Fig. 4:**
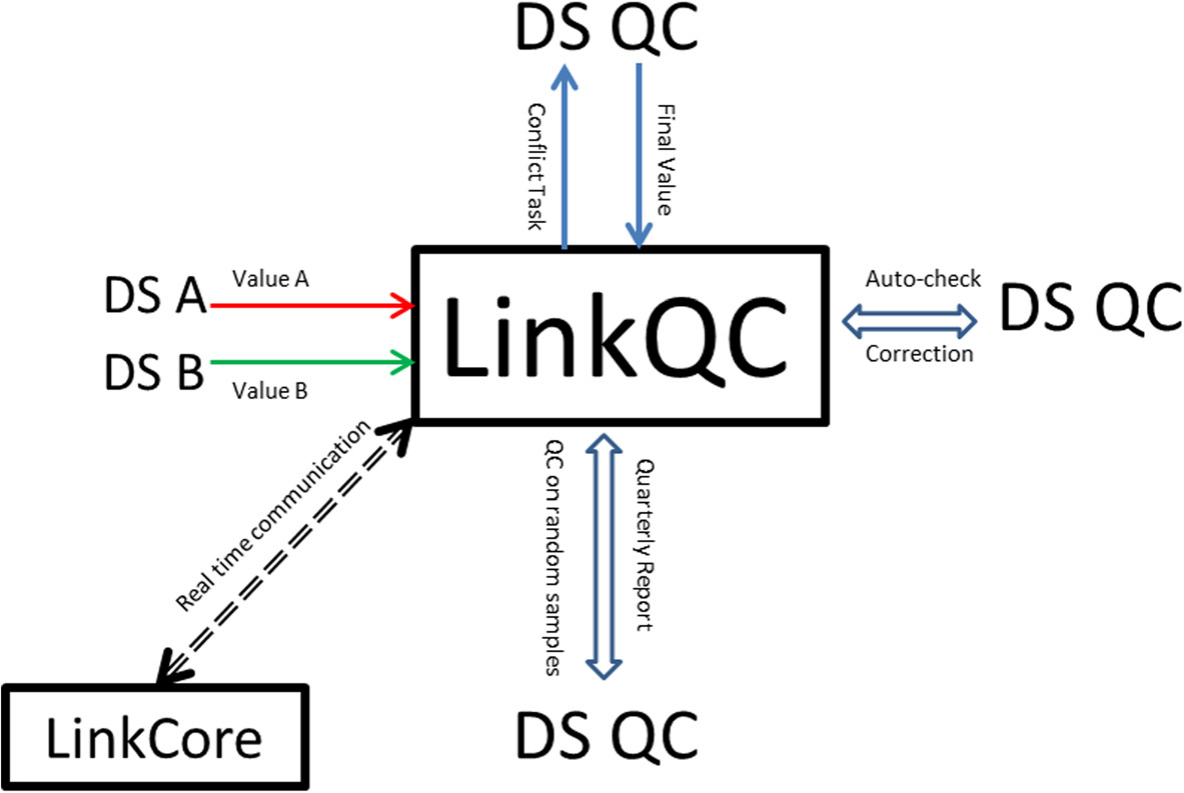
LinkQC system is designed to ensure the quality and integrity of clinical data extracted from medical records

The quality of structured data extracted from complex MRs is of great importance for downstream applications. We design LinkQC to perform quality control (Fig. 4). After receiving entry values from DS A and B, LinkQC executes the comparison function, to detect differences between two entries. Differences, if any, would prompt LinkQC to queue the QC task for DS-QC. Following the manual investigation, DS-QC determines the final values, with iterative feedbacks from DS A and B, and commits them back to LinkQC. The system takes in the final values with specific annotations on QC process. Additionally, LinkQC performs automatic checks on ranges of entered values and inconsistencies of entered values among themselves and with values in the system.

**Fig. 5:**
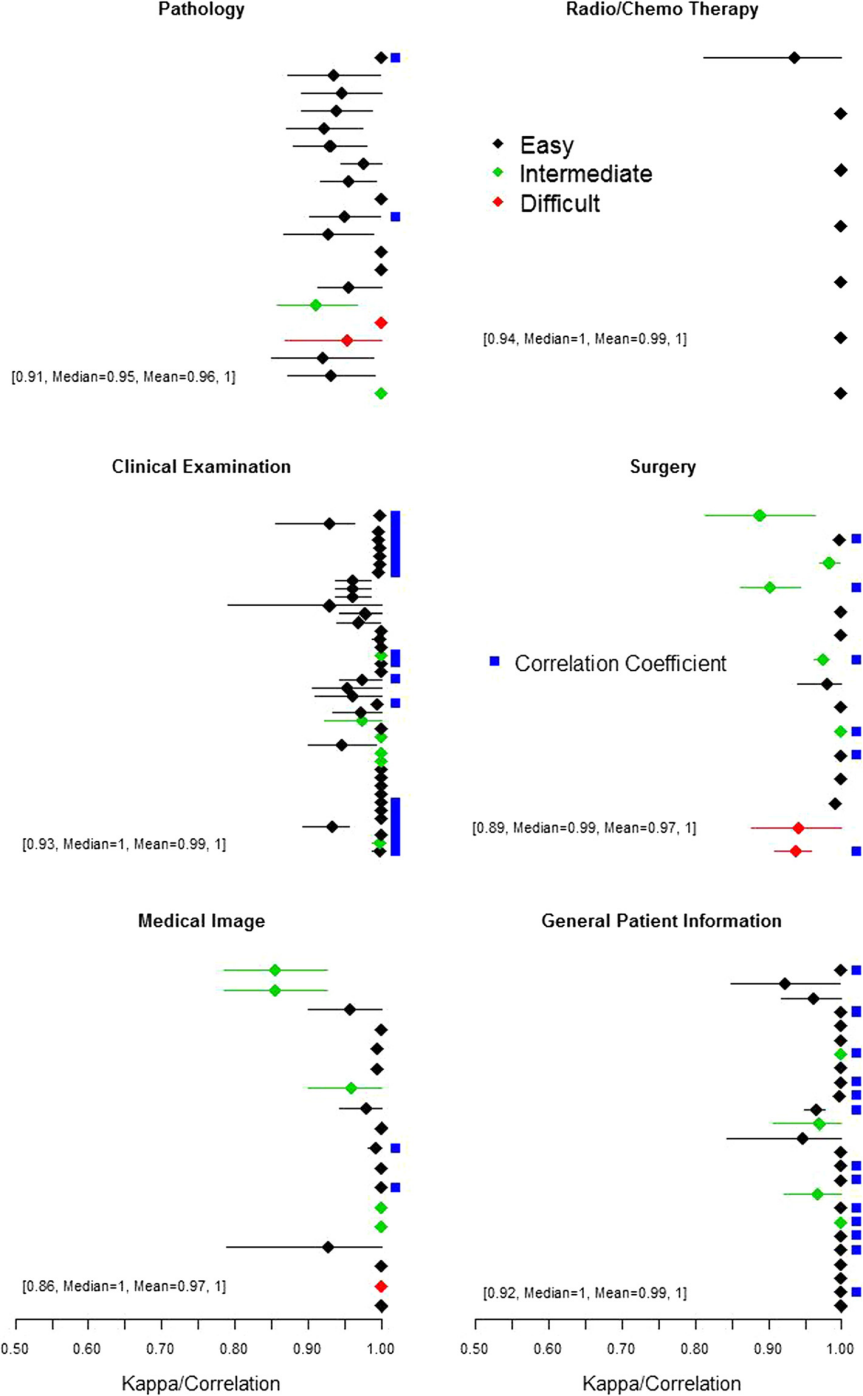
Estimated reproducibility parameters (Kappa statistics for categorical variable and Correlation for continuous variable, denoted by blue square next each row) computed for 127 variables (rows) in six modules (Pathology, Radio/Chemo therapy, Clinical examination, Surgery, Medical image and General patient information). The line denotes 95 % confidence interval. A visual absence of the line implies that very narrow confidence interval. The difficulty level of extracting variable is categorized as easy (black), medium (green) and difficult (red). Within each dot-line plot, a string of texts and numbers provides the range of reproducibility parameters, median and mean, for all variables within the module

LinkQC still needs to cover another gap in quality control. While LinkQC promptly addresses quality control issues due to inconsistent entries, it remains possible that some entry values could be consistently “incorrect”. If extracted data elements are continuous values, or texts, or categorical values with many levels, this concern is minor, since the chance for consistent entries by two independent DS is quite low. However, for categorical variables with only few possible values, e.g., male or female for gender, yes or no for specific procedure, chance consistency can be appreciable. To address this quality concern, LinkQC applies the data range and logic check to detect some obvious errors. Further, LinkQC schedules a sampling-based protocol to ensure the data quality. Specifically, LinkQC takes one random sample of entered data each week in first three weeks of each month, and takes, randomly, $\sqrt{n+1}$ samples (*n* represents the total number of submitted MRs) for the fourth week. On each sample, the current LinkQC protocol repeats the data entry on those error prone variables, to detect possible errors. Finally, to monitor the overall status of quality control, LinkQC documents data quality issues from all sources over time, and assesses if there any temporal unexpected trends in data quality.

### Protection of personal privacy

We design the DRESS system with the following stakeholders in mind: medical directors, physicians, and patients. In this development, we consider personal privacy issues, specifically, medical director’s privacy across different departments, physician’s privacy among physicians, and patient’s privacy from other people who may or may not know the patient. In general, each stakeholder has a password-protected account and each account authorizes access to specific data sets consistent to their assigned roles. Specifically, a medical director has authority to access data created by his/her subordinates on their patients. Each physician has access to his/her patients only, and cannot access patients who receive care by other physicians unless receiving explicit permission. Each patient has access to his/her clinical data only. LinkDoc internally develops this authorization system. It has a dual control on the authorization process: one dimension is on authorized functionalities and the second dimension is on authorized access to data types. Within the first dimension, the system manages users, roles, and scope of authorities. With respect to data access, the system manages which hospital’s data, which department’s data, which data modules within MR and if patient identifier data are involved.

### Data security

Besides protecting personal privacy, DRESS has instituted strict protection of data security throughout data collection and processing. In the front end, LinkMR takes in MRs from clinics. The first task is to extract personal information from MRs, to assign an internal identification numbers, and to store personal information with identification numbers into a separate “linkage data” file, which is known as a LinkData. The second task is to de-identify MR with respect to personal data. The third task is to divide MR into multiple modules. The last task is to encrypt MR images before submitting to the cloud-based data storage managed by LinkCore. Once modulated clinical data enter LinkCore, no one can identify which MR images associate with the corresponding patients, without LinkData, while LinkData is stored in a physically separated storage.

Through implementing above protection strategy, we achieve the following protection goal. First, LinkMR is password-protected, and no one can access the system in the event of losing iPad or PC equipment by HIA. Second, all captured MR images are encryption- protected in the event that unintended parties intercept uploaded data. Third, DRESS has identified multiple roles within the organization with appropriate authorization levels. For example, a junior DS can accesses only his or her data. DS-QC has authority to access those data files where conflicted data values exist. Fourthly, every DS is assigned only one module a time without any reference to other modules or any specific MR, so that it is nearly impossible for any DS to re-identify patients, physicians or hospitals. At the system-wide level, DRESS adopts the state of art technologies to protect the local system with LinkData as well as the database in the cloud storage.

### A reproducibility study

As an enterprise solution, we have implemented the DRESS with over 100 DSs and over 20 DS-QCs. The implemented DRESS is currently processing thousands MRs a day. To assess the actual performance of DRESS, we have randomly sampled 100 MRs from lung cancer patients, whose MRs were entered into the database between October 1, 2015 and December 3, 2015. The only selection criterion is that pathology and surgery modules are not empty. We then manually re-designate these patients’ MRs as new MRs, and repeat the entire DRESS process following the exactly same protocol, finishing data entry within 2 days (December 10 – December 11, 2015). Table 1 shows basic characteristics of these 100 patients, on selected variables. Note that these patients are from multiple hospitals, and their data are used only for evaluating reproducibility of extracted data elements without evaluating any clinical outcomes or addressing any clinical hypotheses. This is deemed as a technology-evaluation study, rather than a clinical study.

**Table 1:** Basic characteristics of 100 patients included in the reproducibility study

Variables	Primary	Repeated	*P*-value
Samples	*n* = 100	*n* = 100	
Gender			
Male	54	54	1.000
Female	46	46	
Age			
<40	5	5	1.000
40-	10	10	
50-	29	29	
60-	56	56	
Drinking			
Never	77	78	1.000
Occasion	13	11	
Current	8	9	
Unknown	2	2	
Smoking			
Never	60	60	1.000
Past	16	16	
Current	23	23	
Unknown	1	1	
ABO Blood Type			
A	29	27	0.992
AB	12	13	
O	31	31	
Unknown	1	0	
Height (cm)			
Mean	165.00	164.00	0.812
SD	8.03	8.02	
Weight (kg)			
Mean	63.40	63.30	0.911
SD	10.72	10.75	
Length of operation (min)			
Mean	167.00	164.00	0.806
SD	64.98	62.86	
Length under anesthesia (min)			
Mean	213.00	209.00	0.738
SD	69.23	66.81	

To be comprehensive in this reproducibility study, we included six modules: pathology, radio/chemo therapy, clinical examination, surgery, medical image and general patient information. Each module includes a set of structured variables (see Additional file 1). There are 217 variables in these modules (Table 2). Based on experiences with DRESS, assessment of some variables is more difficult than other variables, and thus we designate easy, medium and difficult level for each variable. An ease entry means that the value is extractable from one specific position in the MR. The medium level means that DS needs to look through multiple positions in MR for the correct value. The difficult level means that DS needs to look through multiple places in the MR and to synthesize multiple sources of information before making a final decision on the entry value.

**Table 2:** Distribution of selected variables across six modules, after excluding those variables for various reasons

	Selected		Excluded Reasons		
	Variables	Text-based	Too few	Extreme	Other
Pathology	20	12	0	4	2
radio/chemo therapy	7	1	0	0	1
clinical examination	42	4	1	3	1
medical image	18	14	7	8	4
Surgery	15	2	0	6	2
General patients information	25	4	10	2	2
Total	127	37	18	23	12

Included variables are either categorical or continuous. For continuous variables, we use Pearson correlation coefficient to quantify if two independently read values are correlated each other, i.e., using the correlation to quantify their reproducibility. For categorical variables, we use Kappa statistic. Since computation of Kappa statistics is sensitive to the sample size, we include those variables that have at least 50 observations. Further, for categorical variables, we require that none of categorical frequencies exceeds 95 %; otherwise, corresponding Kappa statistics, with exceptional high frequency, are not stable and thus may not be meaningful. After filtering out variables by above criteria, we have 127 variables for reproducibility analysis (Table 2). All statistical analyses and associated table/figure are carried out by R studio, an open source program (https://www.rstudio.com/).

## Results

### DRESS in operation

Based on experiences from building enterprise solutions from Baidu (http://www.baidu.com/), QQ (http://xw.qq.com/) and Alibaba (www.alibaba.com), three of the largest internet companies in China, software engineers at LinkDoc had implemented DRESS, with close collaboration from experienced clinical research staff. As an enterprise solution, DRESS had distinct functions of five departments: 1) training and certification of all DS and DS-QC personnel, 2) implementation of data entry process with multiple subsystems, 3) systematic and iterative quality control, 4) evolving data models to accommodate clinical needs, and 5) continuously monitoring the data entry process to optimize the efficiency and quality of DRESS. We designed the whole enterprise solution in DRESS to allow a team of 50 ~ 1500 full time employees to function effectively, to achieve the economy of the scale while balancing against the volume of data traffic on the system.

### Reproducibility study

Following the reproducibility study protocol, we identified 100 patients who had been diagnosed with lung cancer (see Table 1). Nearly half of patients were male, and most of patients were 60 years or older. Among all patients, 77 patients were non-alcoholic drinkers, and 60 of them were non-smokers. This relatively low percentage of smokers was likely associated with the fact that smoking prevalence among female was less than 1 % in China [32]. Table 1 also listed distribution of ABO blood type, height, weight, length of surgical operation, and length of time under anesthesia, providing an overview of patients included in this reproducibility study.

After retrieving previously entered 100 patient’s MRs, we repeated the double-reading/entry process independently using DRESS. In this process, none of DSs and DS-QCs involved in this study was aware of whether or not he or she had entered a particular module previously, because of large number of daily entry tasks. Distributions of selected variables in the second entry were listed in the second column of Table 1. Differences of their distributions are tested by chi-square test for categorical variables and by t-test for continuous variables, and corresponding *p*-values are listed in the third column of Table 1. Judging from *p*-values and related marginal frequencies/parameters, one would conclude that all selected variables had comparable distributions between two independent entries by DRESS (*p*-value > 0.50).

Investigating reproducibility in details, we computed Kappa statistics for categorical variables and correlation coefficients for continuous variables in all six modules, shown in Fig. 5, while all reproducibility result data are included in Additional file 1. The Pathology module included 20 variables characterizing TNM stages, tissue classifications, immunochemistry, etc.. The overall reproducibility parameters ranged from 0.91 to 1, with median value of 0.95 and mean value of 0.96. There were four continuous variables. Two variables were considered to have medium difficulty in data entry, and two were difficult for data entry. Six variables had nearly perfect reproducibility, while remainders had reproducibility values ranging from 0.80 to 1.0.

The module on radio/chemo therapy included 7 variables, mostly on therapeutic history. All variables were categorical, and were relatively easy for data entry. Other than the history of chemo therapy, other six variables had perfect concordances. The overall concordance ranged from 0.94 to 1.0, with median value of 1 and mean value of 0.99.

The next module was on clinical examination, including 42 variables. Variables included in this module covered various physical examinations, laboratory tests, and medical examinations by instrumentations. Over 50 % of variables were continuous, and hence their reproducibility were measured by correlation coefficients. Other than a few variables ranked with intermediate difficulty in data entry, most were specific and are relatively easy for data entry. In general, the reproducibility parameters ranged from 0.93 to 1, with median value of 1.0 and mean value of 0.99. Most of variables associate with very tight confidence intervals, showing that they were highly concordant with each other.

The surgery module included 15 variables, covering various characteristics of surgical operations. Half of the variables included in this module were continuous. Two variables were difficult for data entry, while five variables were at median difficult level for data entry. Remarkably, reproducibility parameters ranged from 0.89 to 1, with median value of 0.99 and mean value of 0.97.

The fifth module was on medical image, including 18 variables characterizing PET-CT scan, MR scan or X-ray. This module included one difficult variable and five variables with medium difficulty for data entry. The overall range of reproducibility was from 0.86 to 1.0 with median and mean values of 1 and 0.97, respectively. Two noticeable outliers (top two rows), corresponding to determination of vascular tumor thrombus and the number of tumors, appeared to have relatively lower Kappa value of 0.85 (95 % CI = 0.78–0.93).

The last module under consideration contained general inpatient information with 25 variables. Most variables were easy for data entry. The overall reproducibility ranged from 0.92 to 1, with median and mean values of 1 and 0.99, respectively. Variables in this module included medical history, history of hospitalization, behavior risk factors, etc.. Relatively high reproducibility provided confidence in potentially using these variables for risk assessment in the downstream analysis.

### Estimated data entry time

One important factor influencing the feasibility of creating a scalable BBD, was how much time was required to convert one unstructured MR data to a semi-structured data useful for pre-specified clinical research purpose. We had attempted to estimate the data entry time for DRESS one patient record, in the current reproducibility study, but had found a bit challenging. As described above, DRESS represented an enterprise solution to process MRs, from data acquisition from clinics, to organizing data modules, to data reading/entry, to iterative quality control, and then to commit semi-structured data into BBD. The averaged entry time for one DRESS patient was influenced by the enterprise scale, experiences of DS and DS-QC personnel, and, of course, medical specialty and intended objective. In this reproducibility study, we estimated approximately 1168 s to enter all 217 variables in six modules. To break down, it took averaged times 155.62 (SD = 101.64), 11.54(SD = 8.12), 153.84(SD = 150.93), 210.79(SD = 77.52), 336(SD = 223.34) and 217.45(SD = 104.49) seconds to enter pathology, radio/chemo therapy, clinical examination, surgery, medical image and general patient information modules, respectively. By the first sight, it may seem surprising that required data entry time on average is amazingly short. Further investigation suggests that there are at least three factors contributing to this short data entry time. First, some data elements are binary check-box, and require little time for data entry. Second, some laboratory data elements are aggregated together, facilitating data entry. Third, the most important reason is the high efficiency of reading and extracting data from homogeneous modules.

## Discussions

In summary, it is probably not debatable that clinical data from healthcare providers are valuable data source for learning and improving healthcare system. What is less clear is how we obtain meaningful and computable data elements from medical records stored in either EMR or, more challenging, the paper-based legacy system. We argued that manual curations by trained physicians are probably not sustainable because of shortage in physicians and nurses whose primary responsibilities are to provide health care. On the other hand, automatically scanning, reading and extracting structured data elements from MRs by machine learning algorithms remain to be an elusive long-term goal for creating usable databases from BBD. Meanwhile this manuscript describes DRESS as an interim solution to create semi-structured database from MR. Combining human intelligence with information technology, DRESS performs the following tasks: 1) scanning MRs; 2) recognizing manually organized modules via OCR technology; 3) encrypting individual modules for data protection before submitting them to cloud storage; 4) assigning college-trained DS to read and extract necessary structured data; 5) entering structured data to an interim database with double-entry; 6) performing quality control; 7) uploading approved values into final database, following rigorous quality control process, and 8) conducting periodic quality control assessments on all data elements in the database. Using the implemented DRESS, we perform an empirical assessment on its reproducibility. While the reproducibility of data entry varies from variable to variable, and from module to module, the overall reproducibility is around 0.98, approaching the perfect reproducibility of 1.

The fundamental idea underlying DRESS is that it divides the complex MR into a collection of relatively homogeneous modules, so that appropriately trained DS, with no formal training in medicine, can comprehend texts within those modules, and can extract relevant data elements from modules. To maximize efficiency of data extraction and quality of extracted data elements, it is important to develop carefully module-specific guidelines and associated training program for all DS. Training starts from basic module, such as patient’s demographic data, and progresses to more complex module, such as surgical procedures, with appropriate certifications at various levels.

While appreciating strengths of DRESS, we should note one important limitation. As expected, typical MR from clinics includes rich information written by physicians and nurses about the patient. What have been extracted by DRESS represent only portion of clinical data from MR, leaving behind much medical information still in unstructured form. To overcome this limitation, our solution is to organize database system of DRESS, with both structured and unstructured data elements, and to make them available to clinical researchers who want to perform additional research on this semi-structured database. On as-needed basis, we can always go back to this semi-structured database, to extract additional data elements. In a longer run when machine learning algorithms and NLP methods are further advanced and are ready for processing MRs, we will be able to apply these methods to process unstructured data. Hence, the value of this semi-structured database is not limited by what have extracted today.

In the above discussion, we restrict to extract data from MR stored on paper-based legacy system. It should be clear that the DRESS is readily applicable to extract data in EMR. The only modification to DRESS described above is that we need to develop a replacement of LinkMR, interfacing DRESS with existing EMR systems, which eliminates the manual operations of scanning MR and uploading images. For this new system, we need to have stringent requirement on data security and data privacy protection, to minimize “data contamination” between systems.

“Divide and conquer”, the fundamental principle of DRESS, is actually the principle of distributed computing. Taking on this principle, we anticipate several extensions to DRESS in the future. As we all know, the medical resources are unevenly distributed throughout the world. Through using computing cloud, DRESS can effectively reach hospitals anywhere in the world. Further, through distributed manual curations, DRESS allows many groups of DS, from different locations and from different culture and language background, to work together.

While appreciating remarkable reproducibility of data elements in DRESS (Fig. 1), one could challenge the main result by asking why we did not assess reliability of DRESS. In theory, one could estimate reliability by comparing DRESS data elements with those extracted by physicians, if the latter were considered as “gold standard”. Actually, prior to launching this reproducibility study, we attempted this reliability study, asking three young and motivated physicians to participate in a reliability study. Briefly, one physician graduated in year 2012 with MD and Ph.D. and is working in a top tier hospital after spending a year oversea, second physician graduated with MD and Ph.D. and started practice in year 2014, and third physician graduated with MD and started practice in year 2011. We asked each physician to read directly from 50 MR images and to extract 217 variables from six modules. Despite enthusiastic supports, it took them three weeks (2015/11/05-2015/11/26), before completing data reading and entry tasks. All three physicians expressed that data entry tasks are tedious and unrewarding. When assessing reproducibility on extracted data variables between three physicians, we found that the reproducibility is quite poor, approximately 40 ~ 60 % for most variables, many of which are rated to be at “easy level”. Actually, our disappointing results are not too far away from a study in United States [33]. In light of the preliminary nature of this reliability experiment, we were unable to make any general conclusion. This firsthand experience informs us that the feasibility of establishing a “gold standard” with manual reading of MRs by physicians is questionable, since physicians have never been hired to read and extract data from MRs. Better alternatives, for example, clinical trial researchers who are medically trained but are specialized in data standard and extractions, needs to be developed for establishing “good standards”..

Based on our direct experiences, we have shown that DRESS can help to create large usable semi-structured database for hospitals/clinics. One sensitive question is who owns all of clinical data in such a database: healthcare organizations, physicians, or patients? While this question is a question for society to answer, it is our conviction in developing DRESS that patients should ultimately own their MR and extracted data, and physicians should be able to utilize all of clinical data in the database to advance clinical research and improve patient-centered outcomes. Towards this goal, DRESS provides a transparent system so that all stakeholders can benefit from big data for their own causes.

## Conclusions

In this paper, we introduce an innovative enterprise solution, known as double-reading/entry system (DRESS), for extracting structured data elements from unstructured medical records. DRESS has three major subsystems (LinkMR, LinkCore and LinkQC) to facilitate data acquisition from clinics, to de-identify medical record data to ensure data security, to implement double-reading/entry process with quality control, and to facilitate data sharing and research. Through a reproducibility study, we have shown that DRESS has high reproducibility for most of clinical variables. Based on both human intelligence and information technology, DRESS represents a hybrid solution to turn unstructured BBD into semi-structured big data, to fulfill the promise of big data analytics for clinical research and services.

## Additional file

Additional file 1: All variables used in six modules designed for capturing key clinical information from treating lung cancer patients. (DOCX 57 kb)

## Abbreviations

BBD: Big biomedical data
DRESS: Double reading-entry system
DS: Data specialist
EMR: Electronic medical record
HIA: Health information assistant
LinkCore: Linkage Core
LinkData: Linkage data
LinkMR: Linking medical records
LinkQC: Linkage quality control
MR: Medical record
NLP: Natural language processing
OCR: Optical character recognition
