# Supplementary material for: A hybrid solution for extracting structured medical information from unstructured data in medical records via a double-reading/entry system
Source: BMC Med Inform Decis Mak. 2016 Aug 30;16(1):114. doi: 10.1186/s12911-016-0357-5 (PMC5006527; doi:10.1186/s12911-016-0357-5)
Supplement: Supplementary file 1 [file 12911_2016_357_MOESM1_ESM.docx]

Module	Variable	Difficulty	Type	N	Kappa	95% CI
Pathology	Differentiation degree of pathological differentiation	2	Discrete	61	1	1.000,1.000
Pathology	Pathological -TNM stage -M	1	Discrete	213	0.932	0.873,0.991
Pathology	Pathological -TNM stage -N	1	Discrete	213	0.92	0.851,0.989
Pathology	Pathologic tumor thrombus and stump cancer	3	Discrete	213	0.955	0.868,1.000
Pathology	Pathological tumor thrombus and stump - nerve invasion	3	Discrete	213	1	1.000,1.000
Pathology	Pathologic pathologic examination.	2	Discrete	213	0.912	0.857,0.967
Pathology	Pathologic differentiation degree	1	Discrete	213	0.956	0.913,1.000
Pathology	Pathology - section of retained specimens - is	1	Discrete	213	1	1.000,1.000
Pathology	Pathology immunohistochemistry -CEA	1	Discrete	213	1	1.000,1.000
Pathology	Pathology immunohistochemistry -CK7	1	Discrete	213	0.9283	0.867,0.989
Pathology	Pathology immunohistochemistry -Ki67	1	Continuous	213	0.95	0.902,0.999
Pathology	Pathology immunohistochemistry -Naspin_A	1	Discrete	213	1	1.000,1.000
Pathology	Pathology immunohistochemistry -TTF_1	1	Discrete	213	0.956	0.917,0.994
Pathology	Pathology - immunohistochemistry - no	1	Discrete	213	0.977	0.945,1.000
Pathology	Pathology - invasion and location - no	1	Discrete	213	0.931	0.880,0.981
Pathology	Pathology - distant metastases - no	1	Discrete	213	0.923	0.870,0.975
Pathology	Pathologic tumor type no	1	Discrete	213	0.94	0.892,0.987
Pathology	Pathologic tumor type tumor classification	1	Discrete	213	0.946	0.892,1.000
Pathology	Pathologic lymph node dissection and metastasis	1	Discrete	518	0.9353	0.8722,0.9983
Pathology	Pathological lymph node dissection and metastasis	1	Continuous	518	1	1.000,1.000
Radio/Chemo Therapy	Targeted therapy - no	1	Discrete	100	1	1.000,1.000
Radio/Chemo Therapy	Radiation conditions - no	1	Discrete	100	1	1.000,1.000
Radio/Chemo Therapy	History of radiation therapy	1	Discrete	100	1	1.000,1.000
Radio/Chemo Therapy	Chemotherapy - no	1	Discrete	100	1	1.000,1.000
Radio/Chemo Therapy	Biological therapy - no	1	Discrete	100	1	1.000,1.000
Radio/Chemo Therapy	History of chemotherapy - end time	1	Date	104	0.9986	0.9979,0.999
Radio/Chemo Therapy	History of chemotherapy - no	1	Discrete	104	0.936	0.811,1.000
Clinical Examination	Pulmonary function tests -DLCO_SB-	1	Continuous	48	0.998	0.996,0.999
Clinical Examination	Pulmonary function -DLCO_SB-	2	Continuous	48	0.998	0.996,0.999
Clinical Examination	DLCO_SB-proportion	1	Continuous	48	1	1.000,1.000
Clinical Examination	Pulmonary function tests -MVV-	1	Continuous	72	0.933	0.893,0.957
Clinical Examination	FEV_1-proportion	1	Continuous	78	1	1.000,1.000
Clinical Examination	FVC-proportion	1	Continuous	78	1	1.000,1.000
Clinical Examination	FEV_1-FVC-proportion	1	Continuous	79	1	1.000,1.000
Clinical Examination	Ambulatory ECG - results	1	Discrete	100	1	1.000,1.000
Clinical Examination	Ambulatory ECG - time	1	Date	100	1	1.000,1.000
Clinical Examination	Check - ambulatory ECG - no	1	Discrete	100	1	1.000,1.000
Clinical Examination	Pulmonary function - diffusion function	1	Discrete	101	1	1.000,1.000
Clinical Examination	Pulmonary function - ventilation function - mixed disorder	2	Discrete	101	1	1.000,1.000
Clinical Examination	Check lung function - ventilation function - restrictive barriers	2	Discrete	101	1	1.000,1.000
Clinical Examination	Pulmonary function - ventilation function - normal	1	Discrete	101	0.9463	0.9,0.993
Clinical Examination	Pulmonary function - ventilation - obstructive	2	Discrete	101	1	1.000,1.000
Clinical Examination	Examination - pulmonary function - no	1	Discrete	101	1	1.000,1.000
Clinical Examination	Pulmonary function - small airway function - results	2	Discrete	101	0.974	0.923,,1.000
Clinical Examination	Endoscopy - bronchoscopy - results	1	Discrete	105	0.972	0.934,1.000
Clinical Examination	Endoscopy - bronchoscopy - time	1	Continuous	105	0.995	0.987,1.000
Clinical Examination	Endoscopy - bronchoscopy - no	1	Discrete	105	0.962	0.909,1.000
Clinical Examination	Abdominal ultrasound examination results	1	Discrete	108	0.955	0.906,1.000
Clinical Examination	Abdominal ultrasound examination time	1	Continuous	108	0.974	0.943,1.000
Clinical Examination	Abdominal ultrasonography - no	1	Discrete	108	1	1.000,1.000
Clinical Examination	Check heart ultrasound -LVEF	1	Continuous	122	1	1.000,1.000
Clinical Examination	Pulmonary artery systolic blood pressure	2	Continuous	122	1	1.000,1.000
Clinical Examination	Examination - cardiac ultrasound - results	1	Discrete	122	1	1.000,1.000
Clinical Examination	Examination - cardiac ultrasound - time	1	Date	122	0.998	0.995,1.000
Clinical Examination	Examination - cardiac ultrasound - no	1	Discrete	122	1	1.000,1.000
Clinical Examination	Examination - ECG - results	1	Discrete	129	0.969	0.939,0.999
Clinical Examination	Check ECG time	1	Date	129	0.9776	0.944,1.000
Clinical Examination	Examination - ECG - no	1	Discrete	129	0.9292	0.7914,1.000
Clinical Examination	Abdominal ultrasound examination site	1	Discrete	392	0.9617	0.938,0.985
Clinical Examination	Abdominal ultrasound examination site	1	Discrete	392	0.9617	0.938,0.985
Clinical Examination	Abdominal ultrasound examination site	1	Discrete	392	0.9617	0.938,0.985
Clinical Examination	Pulmonary function -MVV-	1	Continuous	72	0.9967	0.9945,0.9979
Clinical Examination	Pulmonary function tests -MVV-	1	Continuous	72	0.9989	0.9983,0.9993
Clinical Examination	Pulmonary function tests -FEV_1-	1	Continuous	78	0.9997	0.9995,0.9998
Clinical Examination	Pulmonary function -FEV_1-	1	Continuous	78	0.9997	0.9995,0.9998
Clinical Examination	Pulmonary function tests -FVC-	1	Continuous	78	0.9974	0.9959,0.9983
Clinical Examination	Pulmonary function -FVC-	1	Continuous	78	0.9975	0.9961,0.9984
Clinical Examination	Pulmonary function -FEV_1_FVC-	1	Continuous	79	0.9294	0.8551,0.9642
Clinical Examination	Pulmonary function tests -FEV_1_FVC-	1	Continuous	79	0.9984	0.9975,0.999

Surgery	Operation condition - length of anesthesia	3	Continuous	100	0.938	0.909,0.958
Surgery	Operation condition operation mode	3	Discrete	100	0.941	0.876,1.000
Surgery	Operation condition - operation name	1	Discrete	100	0.991	0.982,0.999
Surgery	Operative information - postoperative blood transfusion - no	1	Discrete	100	1	1.000,1.000
Surgery	Operation - intraoperative bleeding	1	Continuous	100	0.999	0.998,0.999
Surgery	Intraoperative blood transfusion - red blood cell	2	Continuous	100	1	1.000,1.000
Surgery	Operation - intraoperative death	1	Discrete	100	1	1.000,1.000
Surgery	Operation - intraoperative indicators - end expiratory CO2 peak - unit	1	Discrete	100	0.98	0.940,1.000
Surgery	Operation - intraoperative indicators - end expiratory CO2 peak - results	2	Continuous	100	0.975	0.962,0.983
Surgery	Operation condition - intraoperative indicators - no	1	Discrete	100	1	1.000,1.000
Surgery	Operation - perioperative mortality.	1	Discrete	100	1	1.000,1.000
Surgery	Operation condition - postoperative extubation - lead discharge - thoracic duct drainage	2	Continuous	475	0.9034	0.861,0.944
Surgery	Operation - body temperature - less than 37	2	Discrete	482	0.9834	0.969,0.997
Surgery	Operation condition - long operation	1	Continuous	100	0.997	0.9955,0.9980
Surgery	Operation condition and operation mode	2	Discrete	129	0.8886	0.8138,0.9633
Medical Image	Image -PET_CT- no	1	Discrete	100	1	1.000,1.000
Medical Image	Imaging -PET_CT- tumor related conditions - vascular tumor thrombus	3	Discrete	100	1	1.000,1.000
Medical Image	Image -PET_CT- tumor related cases - the number of tumors - a few	1	Discrete	100	1	1.000,1.000
Medical Image	Imaging -PET_CT- tumor related cases - the number of tumors - there is no calcification	1	Discrete	100	0.928	0.788,1.000
Medical Image	Imaging -PET_CT- tumor related cases - metastasis	2	Discrete	100	1	1.000,1.000
Medical Image	Imaging -PET_CT- tumor related cases - metastasis	2	Discrete	100	1	1.000,1.000
Medical Image	Image scan time	1	Continuous	101	1	1.000,1.000
Medical Image	Image - bone scan - no	1	Discrete	101	1	1.000,1.000
Medical Image	Image -MRI- time	1	Continuous	108	0.9936	0.983,1.000
Medical Image	Image -MRI- no	1	Discrete	108	1	1.000,1.000
Medical Image	Imaging -MRI- tumor associated condition - no	1	Discrete	108	0.9806	0.943,1.000
Medical Image	Imaging -CT- tumor associated conditions - tumor - pathology	2	Discrete	110	0.959	0.901,1.000
Medical Image	Image X-ray time	1	Date	199	0.995	0.987,1.000
Medical Image	Image X-ray time	1	Date	199	0.995	0.987,1.000
Medical Image	Image - X - ray	1	Discrete	199	1	1.000,1.000
Medical Image	Image - X-ray - tumor - associated condition - no	1	Discrete	199	0.958	0.901,1.000
Medical Image	Imaging -CT- tumor related conditions - vascular tumor thrombus	2	Discrete	209	0.8553	0.7849,0.9257
Medical Image	Imaging -CT- tumor related cases - the number of tumors - there is no calcification	2	Discrete	209	0.8553	0.7849,0.9257
General Patient Information	Personal history - past medical history - operative history - operative name	1	Discrete	60	1	1.000,1.000
General Patient Information	Personal history - past medical history - History of surgery - operative time	1	Continuous	60	1	1.000,1.000
General Patient Information	Discharge -ICU observation -ICU observation	1	Discrete	100	1	1.000,1.000
General Patient Information	Discharged condition - two operation - no	1	Discrete	100	1	1.000,1.000
General Patient Information	Discharge status - the cost of hospitalization	1	Continuous	100	1	1.000,1.000
General Patient Information	Discharge - hospitalization expenses	1	Continuous	100	1	1.000,1.000
General Patient Information	Discharge - hospital drug costs	2	Continuous	100	1	1.000,1.000
General Patient Information	Discharge - total hospital costs	1	Continuous	100	1	1.000,1.000
General Patient Information	Personal history - the incidence of symptoms - the onset of symptoms	2	Discrete	100	0.968	0.922,1.000
General Patient Information	Personal history - past medical history - high blood pressure - duration of illness	1	Continuous	100	1	1.000,1.000
General Patient Information	Personal history - past medical history - Diabetes - duration of illness	1	Continuous	100	1	1.000,1.000
General Patient Information	Personal history - past medical history - type of diabetes - type	1	Discrete	100	1	1.000,1.000
General Patient Information	Personal history - previous history of cancer treatment - no	1	Discrete	100	0.9468	0.843,1.000
General Patient Information	Personal history - previous cancer treatment history - tumor name	2	Discrete	100	0.969	0.907,1.000
General Patient Information	Personal history - height	1	Continuous	100	0.966	0.949,0.977
General Patient Information	Personal history - weight	1	Continuous	100	0.997	0.995,0.998
General Patient Information	Personal history - smoking history - length of quitting smoking	1	Continuous	100	1	1.000,1.000
General Patient Information	Personal history - smoking history - smoking types	1	Discrete	100	1	1.000,1.000
General Patient Information	Personal history - the history of smoking - the amount of smoking	2	Continuous	100	1	1.000,1.000
General Patient Information	Personal history - smoking history - unit of smoking	1	Discrete	100	1	1.000,1.000
General Patient Information	Personal history - smoking history - smoking status	1	Discrete	100	1	1.000,1.000
General Patient Information	Personal history - smoking history - length of smoking	1	Continuous	100	1	1.000,1.000
General Patient Information	Personal history - blood type -ABO blood type	1	Discrete	100	0.9623	0.918,1.000
General Patient Information	Personal history - drinking history - drinking status	1	Discrete	100	0.923	0.848,0.998
General Patient Information	Personal history - drinking history - long drinking	1	Continuous	100	1	1.000,1.000
